# Intrapartum pudendal nerve block analgesia and risk of postpartum urinary retention: a cohort study

**DOI:** 10.1007/s00192-021-04768-0

**Published:** 2021-04-16

**Authors:** Åsa Henning Waldum, Anne Catherine Staff, Mirjam Lukasse, Ragnhild Sørum Falk, Ingvil Krarup Sørbye, Anne Flem Jacobsen

**Affiliations:** 1 Division of Obstetrics and Gynecology, Oslo University Hospital, Sognsvannsveien 20, 0372 Oslo, Norway; 2 Faculty of Medicine, University of Oslo, Oslo, Norway; 3 Institute of Health Sciences, Oslo Metropolitan University, Oslo, Norway; 4 Centre for Women’s, Family and Child Health, Faculty of Health and Social Sciences, University of South-Eastern Norway, Kongsberg, Norway; 5 Oslo Centre for Biostatistics and Epidemiology, Oslo University Hospital, Oslo, Norway

**Keywords:** Delivery, Obstetric, Postpartum urinary retention, Pudendal nerve block, Analgesia, Obstetrical

## Abstract

**Introduction and hypothesis:**

Pudendal nerve block analgesia (PNB) is used as pain relief in the final stage of childbirth. We hypothesized that PNB is associated with higher rates of postpartum urinary retention.

**Methods:**

We performed a cohort study among primiparous women with a singleton, cephalic vaginal birth at Oslo University Hospital, Norway. Women receiving PNB were included in the exposed group, while the subsequent woman giving birth without PNB was included in the unexposed group. We compared the likelihood of postpartum urinary retention, defined as catheterization within 3 h after birth. Logistic regression analysis stratified by mode of delivery was performed adjusting for epidural analgesia, episiotomy and birth unit.

**Results:**

Of the 1007 included women, 499 were exposed to PNB and 508 were unexposed. In adjusted analyses, women exposed to PNB did not differ in likelihood of postpartum urinary retention compared to women unexposed to PNB in either spontaneous (odds ratio[OR]: 0.82, 95% confidence interval [CI] 0.55–1.22) or instrumental (OR 1.45, 95% CI 0.89–2.39) births. Furthermore, no differences between the groups were observed with excessive residual urine volume or catheterization after > 3 h.

**Conclusions:**

PNB was associated with neither risk of postpartum urinary retention nor excessive residual urine volume and is therefore unlikely to hamper future bladder function.

**Supplementary Information:**

The online version contains supplementary material available at 10.1007/s00192-021-04768-0.

## Introduction

Postpartum urinary retention (PUR) is a common condition following birth. PUR may lead to short- and long-term complications such as bladder dysfunction, recurrent urinary tract infection and, very rarely, bladder rupture [1, 2]. Reported incidences of PUR vary from 0.05% to 37% [3]. Different PUR definitions may explain the wide range [4] as well as differences in patient populations and obstetric care. The definitions by Yip et al. [5] are frequently used [6, 7]. They defined two distinct definitions: overt PUR (the inability to void at all the first 6 h after birth with the requirement of catheterization), which requires early intervention, and covert PUR (post-void residual urine volume of ≥ 150 ml after spontaneous micturition), which normalizes within 2–5 days in most cases [1, 5, 8]. Consensus is lacking for a definition of acute bladder overdistention [9]. In addition, although women with PUR volumes > 750 ml may need catheterization over an extended time period before normal bladder function restoration [10], there is no agreed retention volume above which there is a high risk for irreversible long-term bladder dysfunction.

The known risk factors for urinary retention include advanced age, nerve dysfunction and bladder outlet obstruction [11]. Among obstetric risk factors for PUR are epidural analgesia, primiparity, instrumental vaginal birth and episiotomy [12–14]. The mechanisms for PUR thus include mechanical, functional and neurological factors, and likely vary among women and delivery settings.

Voluntary control of the lower urinary tract requires interaction between autonomic (sympathetic and parasympathetic nerves) and somatic, afferent (sensory nerve) and efferent (motor nerve) pathways. Somatic efferent function deterioration leads to an attenuated contractile force with insufficient urine evacuation as a result [11]. The pudendal nerve has 30% autonomic and 70% somatic fibers and innervates the lower third of the vagina, urethra and perineum. The autonomic fibers in the pudendal nerve transmit the sensation of necessity to void [15]. It is possible that neural analgesia during birth could lead to attenuated contractile force, and reduced transmission in the autonomic fibers could thereby reduce the necessity to void.

During the first stage of birth (from 3 to 4 cm to 10 cm dilatation), the most efficient pain relief is epidural analgesia, whereas pudendal nerve block (PNB) is an option during the second stage of birth (from 10 cm to birth) during the final descent of the fetal head and expulsion of the baby. PNB is an effective method of pain relief, providing analgesia to the vulva and anus [16–18] by transvaginal infiltration of the pudendal nerve. It may be provided in spontaneous as well as in instrumental (vacuum and/or forceps extraction) vaginal births. PNB may also be used as analgesia for the suturing of perineal lacerations after birth. The use of PNB in Norway and Sweden has decreased since the 1980s [19, 20], probably because of the increased availability of epidural analgesia [20]. Known adverse effects of PNB are few, but include a slight transient decline in uterine activity [18] as well as a reduction of the bearing down reflex [19], especially when epinephrine is added. Case studies have described the occurrence of hematoma [21] and abscess [22] and interference with the newborn breast-seeking behavior after births where PNB was provided [23]. However, to our knowledge, no prior studies have investigated the association between PNB and risk of PUR. In this study we hypothesized that PNB was positively associated with overt PUR. In addition, we explored the association between PNB and secondary outcomes, including excessive residual urine volume when overt PUR was diagnosed, catheterization after more than 3 h postpartum, Apgar score and anal sphincter injury after stratification by mode of delivery.

## Materials and methods

### Study design and population

This study was conducted at the Department of Obstetrics at Oslo University Hospital (OUH). OUH has two delivery locations: Rikshospitalet with 2500 births/year (named Unit 1) and Ullevål with 6950 births/year (named Unit 2). The two units share clinical guidelines and management. Unit 1 reintroduced PNB as part of quality improvement in 2014, aiming to ensure uniform pain relief availability for all women. In this unit, women with a request for more pain relief may be provided PNB regardless of whether the midwife alone, or in collaboration with the attending doctor, handles the birth. Primiparous women having delivered a singleton live newborn in cephalic position at term, during the period from January 1, 2017, until June 1, 2019, were eligible for inclusion. All women who were exposed to PNB during birth were invited to participate (pudendal nerve block group). For each invited woman exposed to PNB, the subsequent woman at the same unit unexposed to PNB was invited to participate (non-pudendal nerve block group). The eligible women received written study information 4 weeks postpartum. The women were informed that a response to the attached questionnaire about childbirth experiences was considered a written informed consent. The childbirth experience is the focus of an ongoing study. In accordance with patient consent, we collected clinical data from the electronic medical records. In addition, we thoroughly reviewed the electronic medical records for reported hematoma or abscess.

We excluded women transferred during birth from the low-risk midwife-led birth unit (with no medical pain relief available), women with allergy to local anesthetics and women with uncertainty of timing (before or after birth) or status (categorization) of PNB.

This study was originally designed to explore the association between PNB and childbirth experience aimed at including 1000 women. With the same sample size available, we may detect an absolute difference of 10% in overt PUR among women exposed compared to unexposed to PNB among women with spontaneous births, assuming a baseline rate of 25%, a power of 80% and a significance level of 5%. We considered a difference of 10% in overt PUR to be clinically relevant. For instrumental deliveries the available sample was smaller, and thus the power was < 80%.

### Variables

The exposure in this study was PNB provided during birth, whereas overt PUR was the primary outcome. We defined overt PUR as the inability to void within 3 h postpartum, equaling the need for at least one catheterization within 3 h postpartum according to the department’s guideline.

We considered the proportions of two postpartum catheterization volumes, namely ≥ 750 ml and ≥ 1000 ml. Other secondary outcomes were catheterizations after > 3 h postpartum, Apgar score < 7 at 5 min of age and anal sphincter injury.

Clinical data included maternal age at birth (years), marital status (married/cohabiting or not), higher maternal education (> 13 years or not), body mass index (kg/m^2^), gestational length at delivery (days) and birth weight (grams). Epidural analgesia, spinal analgesia, instrumental birth (vacuum and/or forceps extraction), episiotomy and oxytocin used during birth were dichotomous variables. Prolonged second stage of birth was defined as > 3 h and long duration of birth as > 12 h (start of birth defined as 3–4 cm dilation and with regular contractions). Local anesthetics used in PNB were bupivacaine, lidocaine or bupivacaine with epinephrine. PNB duration was defined in minutes from administration to the birth of the baby. Birth unit was either Unit 1 (Rikshospitalet) or Unit 2 (Ullevål).

### Statistical analyses

Descriptive analyses were presented as frequencies and proportions for categorical variables and mean with standard deviation (SD) for normally distributed continuous variables. Comparison of demographic and obstetric characteristics between the groups exposed and unexposed to PNB was performed by chi-square test, Fisher’s exact test and t-test, as appropriate. Primary and secondary outcomes are presented as numbers and proportions with 95% confidence intervals (CI) according to group. The association between PNB and instrumental delivery is complex, and sometimes the first is chosen when planning the latter, and therefore we chose to stratify by mode of birth (spontaneous or instrumental) in our analysis of PNB’s association with PUR.

We explored the association between PNB and overt PUR by performing logistic regression analysis, stratified by mode of birth. Both unadjusted and adjusted odds ratios (aOR) with 95% CI are presented. Due to the restricted number of observations in the stratified analysis, four variables were included in the multivariable analyses. Adjustments were made for epidural and/or spinal analgesia, episiotomy and birth unit, as these were pre-defined based on a potential association with PNB and overt PUR. During birth, epidural (continuous infusion) and spinal (single injection) analgesia are provided in the same anatomical segments, and we therefore merged epidural and spinal analgesia for the purpose of adjustment in the regression analyses. Prolonged birth [both total duration of birth (> 12 h) and prolonged second stage of birth (> 3 h)] was strongly correlated to epidural analgesia and thus omitted from the analyses. Interaction effects between the adjusting variables and PNB were tested by adding product terms, one at a time, into the models.

We used the same logistic regression frameworks for the secondary outcomes, residual urine volume ≥ 750 among women with overt PUR and catheterization after > 3 h. Furthermore, in a sensitivity analysis, missing information about residual volume was considered as < 750 ml if no value was registered, based on the assumption that lack of registrations was likely lack of a large volume. Because of few observations of residual urine volume ≥ 1000 ml when overt PUR was diagnosed, Apgar score < 7 at 5 min of age and sphincter injury, we did not perform multivariate analyses on these secondary outcomes.

We considered an association with a *p* value < 0.05 as statistically significant. We conducted all analyses using SPSS version 25 (IBM Corp., Armonk, NY, USA).

### Ethical approval

All enrolled women provided informed consent. The local Data Personal Officer at OUH approved the study, 2016/18884. This quality study was exempt from approval by the Regional Committee for Medical and Health Research Ethics system in Norway according to the Norwegian act on health research. The study is registered in www.clinicaltrials.gov (NCT04391075). The user council of the Division of Obstetrics and Gynecology at Oslo University Hospital was involved in the design and the development of the study.

## Results

**Fig. 1:**
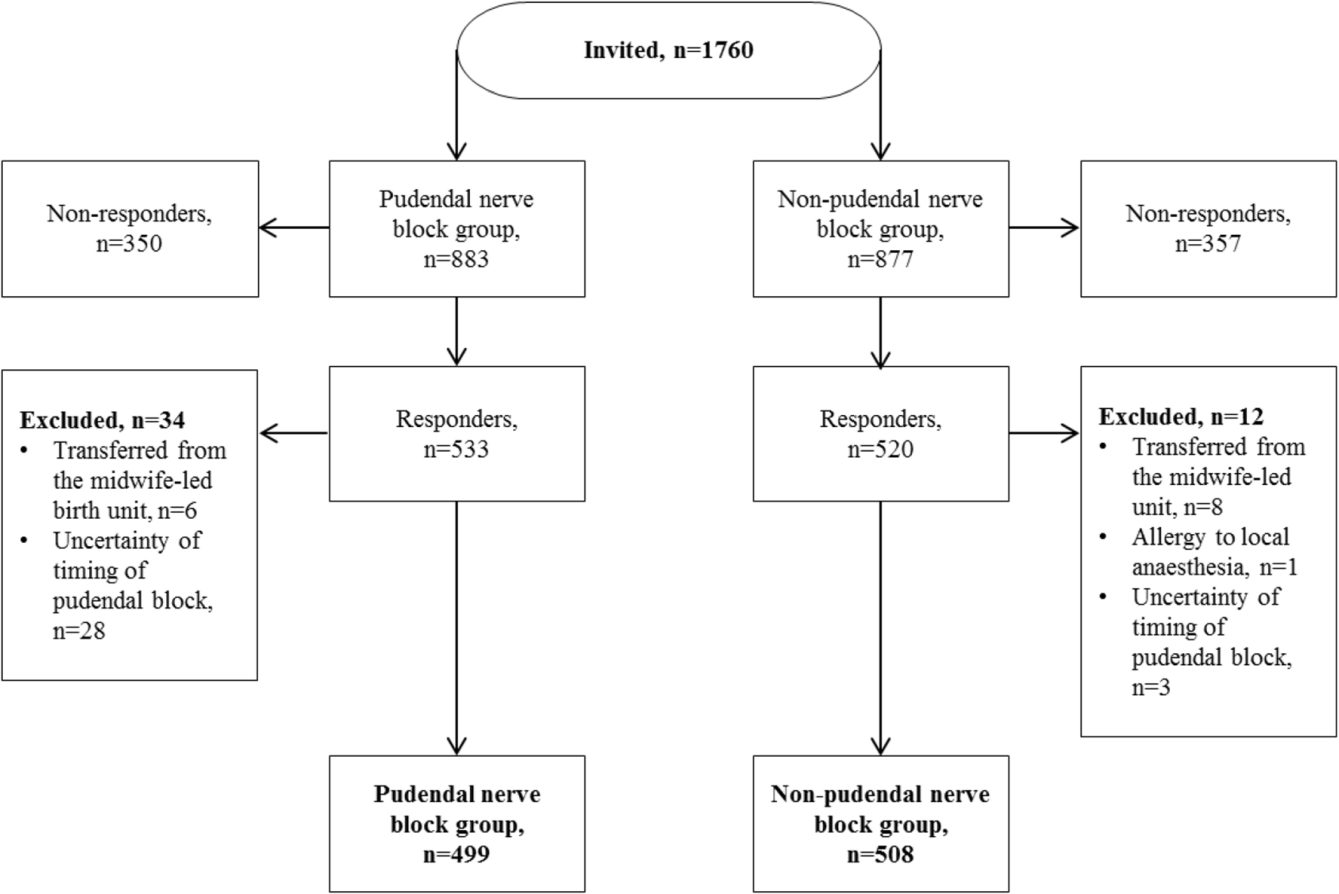
Flow diagram of the inclusion process

A total of 1760 primiparous women were invited to participate in the study. The final study sample comprised 1007 women, 499 women exposed to PNB and 508 women not exposed to PNB. In the PNB group 46% had an instrumental birth, whereas the proportion was 20% in the non-PNB group, while the proportion of overt PUR was 36% (PNB group) and 30% (non-PNB group) (Fig. 1). Adjusted analyses did not show any significant association between PNB and overt PUR in either spontaneous (aOR 0.82, 95% CI 0.55–1.22) or instrumental (aOR 1.45, 95% CI 0.89–2.39) births (Table 3).

Adjusted analyses of PNB and the secondary outcomes residual urine volume ≥ 750 ml and catheterization after > 3 h did not show any significant difference between the groups in either spontaneous or instrumental births (Tables 4 and 5).

Maternal age, married/cohabiting status, education level and body mass index did not differ between the groups. Women exposed to PNB had 2 days longer gestational length at delivery and slightly larger babies compared to women unexposed to PNB. The use of epidural and spinal analgesia did not differ between groups, but women exposed to PNB had higher proportions of instrumental birth and episiotomy. Women exposed to PNB had higher rates of prolonged second stage of birth and long duration of birth. The majority of women in the PNB group were provided PNB with bupivacaine, with doses of 25 mg on both sides (data not shown). There was no difference in newborn rates of Apgar score < 7 at 5 min of age between the groups. The anal sphincter injury rate was low overall, without any difference between the groups (Table 1).

**Table 1:** Demographic and obstetric characteristics of the two study groups, delivering women exposed and unexposed to pudendal block (*n* = 1007)

	Pudendal nerve block group (*n* = 499) Mean ± SD or *n* (%)	Non-Pudendal nerve block group (*n* = 508) Mean ± SD or *n* (%)	*p* value
Maternal age (years)	32.1 ± 4.2	32.2 ± 4.0	0.61
Married/cohabiting*	475 (96.3)	476 (94.1)	0.09
Higher education *Missing*	420 (93.5) *50*	412 (92.6) *63*	0.57
Body mass index (km/m²) Missing	23.3 (4.3) *92*	22.9 (4.0) *100*	0.25
Gestational length (days)	282 ± 7.9	280 ± 8.6	< 0.001
Birth weight (grams)	3538 ± 468	3416 ± 426	< 0.001
Epidural analgesia	332 (66.5)	364 (71.7)	0.08
Spinal analgesia	24 (4.8)	13 (2.6)	0.06
Epidural and/or spinal analgesia	350 (70.1)	374 (73.6)	0.22
Instrumental birth^1^	231 (46.3)	102 (20.1)	< 0.001
Episiotomy*	285 (58.5)	195 (39.8)	< 0.001
Prolonged second stage of birth (> 3 h)*	117 (23.5)	63 (12.4)	< 0.001
Long duration of birth (> 12 h)*	105 (21.2)	65 (12.8)	< 0.001
Oxytocin augmentation*	322 (64.7)	290 (57.1)	0.01
Pudendal block anesthetic			
Bupivacaine	357 (85.4)	–	–
Lidocaine	50 (12.0)	–	–
Bupivacaine with epinephrine	11 (2.6)	–	–
Missing	81	–	–
Pudendal block duration (minutes)	67 ± 61	–	–
Birth unit			
Unit 1 (Rikshospitalet)	328 (65.7)	338 (66.5)	0.79
Unit 2 (Ullevål)	171 (34.3)	170 (33.5)	
Primary outcome			
Overt PUR^2^ *Missing*	180 (36.8) *10*	147 (29.6) *12*	0.02
Secondary outcomes			
Residual urine volume when overt PUR was diagnosed			
≥ 1000 ml^3^ *Missing*	13 (9.3) *40*	22 (17.5) *21*	0.05
≥ 750^3^ *Missing*	42 (30.0) *40*	40 (31.7) *21*	0.76
Volume, ml^3^ (range) *Missing*	575 ± 330 (30–1700) *40*	597 ± 360 (100–1600) *21*	0.60
Catheterized after > 3 h postpartum*	69 (14.0)	47 (9.3)	0.02
Apgar score < 7 at 5 min of age	7 (1.4)	7 (1.4)	0.97
Anal sphincter injury *Missing*	8 (1.7) *25*	6 (1.2) *26*	0.57

Missing data are presented as a separate category if ≥ 5%, marked (*) if < 5%, otherwise variable information was complete. ^1^Vacuum and/or forceps extraction. ^2^Overt postpartum urinary retention (PUR): catheterization within 3 h postpartum. ^3^Among women diagnosed with overt PUR. ^4^T-test for continuous variables and Pearson chi-square for categorical variables

When stratifying also for mode of birth, differences between women exposed and unexposed to PNB varied similarly to those shown in Table 1. However, variations were mostly in the spontaneous birth group, while women with instrumental birth varied less between groups exposed to PNB or not (Supplementary Table 1).

In this cohort, there were some differences in obstetric characteristics between the units: Unit 1 had shorter PNB duration than Unit 2. Unit 1 had more overt PUR and more epidural and spinal analgesia, but the rates of episiotomy and instrumental birth did not differ (Supplementary Table 2).

Women with instrumental births had more frequent overt PUR (51% vs. 24%) and were more often catheterized after > 3 h (18% vs. 9%) compared to women with spontaneous birth. The rates of excessive residual urine volume (≥ 1000 or ≥ 750) did not differ with mode of delivery (Table 2). We did not observe any statistically significant differences in overt PUR, high rates of excessive residual urine or catheterization after > 3 h between women exposed and unexposed to PNB in either mode of birth. The rate of residual urine volume ≥ 750 ml did not differ among groups. Imputing missing volume information did not alter the results (data not shown). The rates of newborn Apgar score < 7 at 5 min of age and anal sphincter injury were < 3% in both groups and strata and without any statistically significant differences between the groups (Table 2).

**Table 2:** Primary (overt postpartum urinary retention) and secondary outcomes (overt postpartum urinary retention, catheterization, Apgar score and anal sphincter injury) for the two study groups (delivering women exposed or unexposed to pudendal nerve block) stratified for mode of birth (spontaneous or instrumental)

Spontaneous birth *n* = 674													
	All *n = 674*				Pudendal nerve block group *n* = 268				Non-pudendal nerve block group *n* = 406				*p* value
	***N***^*3*^	***n***^*3*^	***%***	***95% CI***	**N**^3^	**n**^3^	**%**	**95% CI**	**N**^3^	**n**^3^	**%**	**95% CI**	
Overt PUR^1^	*657*	*159*	*24.2*	*21.0–27.7*	260	57	21.9	17.1–27.5	397	102	25.7	21.5–30.3	0.27*
Residual urine ≥ 1000 ml when overt PUR was diagnosed	*138*	*17*	*12.3*	*7.3–19.0*	50	3	6.0	1.3–16.6	88	14	15.9	9.0–25.2	0.09*
Residual urine ≥ 750 ml when overt PUR was diagnosed	*138*	*43*	*31.2*	*23.6–39.6*	50	15	30.0	17.9–44.6	88	28	31.8	22.3–42.6	0.83*
Catheterized after > 3 h postpartum	*672*	*58*	*8.6*	*6.6–11.0*	267	26	9.7	6.5–13.9	405	32	7.9	5.5–11.0	0.41*
Apgar score at 5 min of age < 7	*674*	*5*	*0.7*	*0.2–1.7*	268	1	0.4	0.0–2.1	406	4	1.0	0.3–2.5	0.65**
Anal sphincter injury	*644*	*7*	*1.1*	*0.4–2.2*	258	2	0.8	0.1–2.7	386	5	1.3	0.4–3.0	0.71**
Instrumental birth^2^ ***n*** = 333													
	All ***n = 333***				Pudendal nerve block group ***n*** **= 231**				Non-pudendal nerve block group ***n*** **= 102**				***p*** **value**
	***N***^*3*^	***n***^*3*^	***%***	***95% CI***	**N**^3^	**n**^3^	**%**	**95% CI**	**N**^3^	**n**^3^	**%**	**95% CI**	
Overt PUR^1^	*328*	*168*	*51.2*	*45.7–56.8*	229	123	53.7	47.0–60.3	99	45	45.5	35.4–55.8	0.17*
Residual urine ≥ 1000 ml when overt PUR was diagnosed	*128*	*18*	*14.1*	*8.6–21.3*	90	10	11.1	5.5–19.5	38	8	21.1	9.3–37.3	0.14*
Residual urine ≥ 750 ml when overt PUR was diagnosed	*128*	*39*	*30.5*	*22.6–39.2*	90	27	30.0	20.8–40.6	38	12	31.6	18.5–48.7	0.86*
Catheterized after > 3 h postpartum	*327*	*58*	*17.7*	*13.8–22.3*	227	43	18.9	14.1–24.7	100	15	15.0	8.6–23.5	0.39*
Apgar score at 5 min of age < 7	*333*	*9*	*2.7*	*1.2–5.1*	231	6	2.6	1.0–5.6	102	3	2.9	0.6–8.4	1.00**
Anal sphincter injury	*312*	*7*	*2.2*	*0.9–4.6*	216	6	2.8	1.0–5.6	96	1	1.0	0.0–5.7	0.68**

^1^Catheterization within 3 h postpartum. ^2^Vacuum and/or forceps extraction ^3^*N* = numbers of women with valid information. *n* = number of women the presence of the given characteristics. CI = confidence interval. PUR = postpartum urinary retention. *Pearson Chi-square, **Fisher’s exact test

We observed an interaction effect between PNB and birth unit in the spontaneous birth group (*p* = 0.03) (data not shown). Thus, stratified analyses by birth unit were performed in spontaneous birth (Supplementary Table 3). None of these analyses showed any statistically significant association between PNB and overt PUR. Finally, no case of hematoma or abscess was registered (in the electronic medical records) for any of the participating women (Tables 3, 4, and 5).

**Table 3:** Odds ratios of factors associated with overt postpartum urinary retention, stratified by mode of birth (spontaneous or instrumental)

Spontaneous birth *n* = 630^3^								
	Unadjusted OR	95% CI		*p* value	Adjusted OR^4^	95% CI		*p* value
Pudendal nerve block	0.81	0.56	1.18	0.27	0.82	0.55	1.22	0.33
Epidural/spinal analgesia	2.26	1.49	3.41	< 0.001	2.06	1.34	3.18	< 0.01
Episiotomy	1.30	0.87	1.93	0.20	1.34	0.89	2.02	0.16
Birth unit^1^	2.98	1.92	4.64	< 0.001	2.68	1.71	4.21	< 0.001
Instrumental birth^2^ ***n*** = 326^3^								
	Unadjusted OR	95% CI		*p* value	Adjusted OR^4^	95% CI		*p* value
Pudendal nerve block	1.39	0.87	2.24	0.17	1.45	0.89	2.39	0.14
Epidural/ spinal analgesia	2.76	1.35	5.64	< 0.01	2.79	1.30	5.95	< 0.01
Episiotomy	1.13	0.54	2.38	0.74	1.29	0.60	2.75	0.52
Birth unit^1^	2.53	1.55	4.13	< 0.001	2.30	1.40	3.80	< 0.01

^1^Unit 1 (Rikshospitalet) compared with Unit 2 (Ullevål). ^2^Vacuum and/or forceps extraction. ^3^Number of women included in the analysis. ^4^Multivariable model, epidural/spinal analgesia, episiotomy and birth unit were included. OR: odds ratio; CI: confidence interval

**Table 4:** Odds ratios of factors associated with residual volume ≥ 750 ml, stratified by mode of birth (spontaneous or instrumental)

Spontaneous birth ***n*** = 132^3^								
	Unadjusted OR	95% CI		*p* value	Adjusted OR^4^	95% CI		*p* value
Pudendal nerve block	0.92	0.43	1.95	0.83	0.81	0.36	1.81	0.61
Epidural/spinal analgesia	2.09	0.78	5.55	0.14	2.06	0.75	5.67	0.16
Episiotomy	0.52	0.23	1.18	0.12	0.49	0.21	1.13	0.10
Birth unit^1^	0.55	0.21	1.42	0.21	0.47	0.17	1.28	0.14
Instrumental birth^2^ ***n*** = 128^3^								
	Unadjusted OR	95% CI		*p* value	Adjusted OR^4^	95% CI		*p* value
Pudendal nerve block	0.93	0.41	2.11	0.86	0.94	0.41	2.16	0.88
Epidural/ spinal analgesia	1.34	0.26	6.94	0.73	1.79	0.32	10.05	0.51
Episiotomy	1.11	0.33	3.77	0.87	1.11	0.32	3.85	0.87
Birth unit^1^	0.50	0.20	1.26	0.14	0.46	0.18	1.20	0.11

^1^Unit 1 (Rikshospitalet) compared with Unit 2 (Ullevål). ^2^Vacuum and/or forceps extraction. ^3^Number of women included in the analysis. ^4^Multivariable model, epidural/spinal analgesia, episiotomy and birth unit were included. OR: odds ratio; CI: confidence interval

**Table 5:** Pudendal nerve block and secondary outcome catheterization > 3 h postpartum, stratified by vaginal mode of birth (spontaneous or instrumental)

Spontaneous birth ***n*** = 644^3^								
	Unadjusted OR	95% CI		*p* value	Adjusted OR^4^	95% CI		*p* value
Pudendal nerve block	1.26	0.73	2.16	0.41	1.45	0.83	2.54	0.19
Epidural/ spinal analgesia	1.67	0.91	3.07	0.10	1.60	0.84	1.04	0.15
Episiotomy	1.49	0.84	2.64	0.18	1.49	0.83	2.68	0.18
Birth unit^1^	2.88	1.43	5.79	< 0.01	2.74	1.34	5.58	< 0.01
Instrumental birth^2^ ***n*** = 325^3^								
	Unadjusted OR	95% CI		*p* value	Adjusted OR^4^	95% CI		*p* value
Pudendal nerve block	1.32	0.70	2.52	0.39	1.27	0.66	2.43	0.48
Epidural/spinal analgesia	1.21	0.48	3.04	0.68	1.30	0.47	3.56	0.62
Episiotomy	1.12	0.41	3.05	0.83	1.20	0.44	3.31	0.72
Birth unit^1^	2.37	1.15	4.91	0.02	2.26	1.08	4.72	0.03

^1^Unit 1 (Rikshospitalet) compared with Unit 2 (Ullevål). ^2^Vacuum and/or forceps extraction. ^3^Number of women included in the analysis. ^4^Multivariable model, epidural/spinal analgesia, episiotomy and birth unit were included. OR: odds ratio; CI: confidence interval

## Discussion

In this cohort study of 1007 primiparous women we did not find any significant association between PNB and overt PUR (i.e., catheterization < 3 h postpartum) in adjusted analyses stratified by mode of birth. Nor did we find any significant association between PNB and rates of excessive residual urine volume or between PNB and rate of catheterization after > 3 h postpartum. No adverse effects of PNB on newborn (Apgar score) or maternal (abscess or hematoma) outcomes were observed. There was a difference in frequency of postpartum catheterization between the units despite their shared management and guidelines. The lacking association between PNB and overt PUR was however not affected by this difference.

To our knowledge, the association between PNB and postpartum urinary retention has not been investigated and published previously. Only one previous study on hemorroidectomy in both women and men (*n* = 163) investigated the association between PNB and urinary retention [24]. They found that voiding was less challenging after PNB compared to spinal analgesia (8% vs. 70% urinary retention). In our study, the prevalence of overt PUR was 37% in women exposed to PNB and 30% in women unexposed to PNB, which is relatively high compared to previously reported incidences of PUR varying from 0.05% to 37% [3]. The cause of PUR after vaginal birth is likely multifactorial, including known risk factors such as primiparity, instrumental birth, episiotomy and epidural analgesia. It is also likely that bladder outlet obstruction (caused by swollen mucosa), nerve complication (caused by the passage of the fetus through the birth canal) and a reduced sensation of necessity to void due to analgesia could represent additional risk [11].

A previous systematic review identified an 8% increased risk of PUR in laboring women with epidural analgesia [12]. If this is due to a reduced sensation of necessity to void, a similar association could be present for PNB. However, our study did not reveal an independent association between PNB and overt PUR.

First, our results of a lacking association between PNB and overt PUR may be explained by the position of PNB as a more peripheral analgesia than epidural analgesia, thereby resulting in less neurologic complications. Peripheral nerve blocks have previously been shown to induce less urinary retention than epidural analgesia after knee surgery [25, 26]. The comparison of a central or peripheral analgesia and urinary function after childbirth has to our knowledge not been previously studied.

Second, our finding of a lack of association between PNB and overt PUR may be partly explained by the clinical practice of pre-emptying the bladder prior to instrumental birth, which may have led to a lower urine volume in the bladder after birth. We stratified for mode of birth, and the lack of increased PUR in both instrumental and spontaneous delivery with PNB supports our conclusion of no association between PNB and overt PUR in this cohort.

Third, the small proportion of residual urine volume ≥ 1000 ml in each group restricts the applicability of adjusted analyses. On the other hand, we did not identify any association between PNB and residual urine volume ≥ 750 ml in adjusted regression analyses, strengthening the probability that there is no impact of PNB on the rate of excessive residual urine volumes.

In our study we showed a relatively large proportion of overt PUR (37% and 30% in women exposed an unexposed to PNB); this is likely due to our definition of overt PUR (i.e., catheterization < 3 h postpartum), which is a shorter time than that of Yip et al. [5]. Yip et al. showed an incidence of overt PUR of 4.9%. Our definition is based on the departmental protocols advising catheterization if the woman has not voided within 3 h postpartum in order to avoid large residual urine volumes. In the future, this proactive approach to catheterization in our department could possibly be supplemented by more extensive use of ultrasound measurement of residual volumes in order to avoid unnecessary catheterization of smaller volumes.

The strengths of this study include the prospective design and the large sample size. Another strength is the adjustment for obstetric risk factors of PUR [12], epidural analgesia, episiotomy and instrumental vaginal birth. Primiparity is a risk factor for PUR [12], and including primiparous women only excludes parity as a confounder in this study. This limits the generalization to multiparous women who have a lower risk of PUR. However, our results can be generalized to primiparous women in similar settings and probably to other high income countries. We were not permitted to record data from non-responders. However, data from the Medical Birth Registry of Norway demonstrate that our cohort is similar to the total primiparous group of women in Oslo regarding age (mean 32 years) and being married/cohabiting (94%)) [27], suggesting a representative cohort sample.

Our data are observational and cannot demonstrate causality. We considered a randomized clinical trial but found it unethical to randomize women to pain relief or placebo during birth. Furthermore, bias due to unknown and unmeasured confounders may be another limitation of observational studies such as ours.

Even though overt PUR may be of multifactorial origin, the results of our study clearly show that PNB is not associated with an increased risk of postpartum urinary retention or excessive residual urine volume after spontaneous vaginal birth. To evaluate the safety concerning PUR in instrumental vaginal births, low Apgar score and anal sphincter injury, a larger sample size is required because of the low incidence, but we found no indication of such risks.

In conclusion, we suggest the results of our study can provide women with a better basis for making informed choices regarding the use of PNB as pain relief during birth. Whether PNB provides a better overall childbirth experience is yet to be determined, but it has been shown to provide good pain relief during birth and for suturing lacerations after birth [19]. Our study supports that PNB may be used as a supplementary analgesic method in the second stage of birth without increased risk of overt PUR and excessive residual urine volume and that PNB is thus unlikely to hamper future bladder function.

## Supplementary Information

ESM 1 (DOCX 30 kb)

## Abbreviations

PNB: Pudendal nerve block analgesia
PUR: Postpartum urinary retention
OUH: Oslo University Hospital
