# Supplementary material for: Intrapartum pudendal nerve block analgesia and risk of postpartum urinary retention: a cohort study
Source: Int Urogynecol J. 2021 Apr 16;32(9):2383–91. doi: 10.1007/s00192-021-04768-0 (PMC8418588; doi:10.1007/s00192-021-04768-0)
Supplement: Supplementary file 1 — (DOCX 30 kb) [file 192_2021_4768_MOESM1_ESM.docx]

**Supplementary material**

*Intrapartum pudendal nerve block analgesia and risk of postpartum urinary retention: a cohort study*

*Waldum et al.*

Supplementary table 1. Characteristics of the pudendal block and non-pudendal block groups in women with spontaneous or instrumental vaginal birth, n=1007

|  | **All** | **Spontaneous vaginal birth**  **N=674** | | | **Instrumental vaginal birth**  **N=333** | | |
| --- | --- | --- | --- | --- | --- | --- | --- |
|  | n=1007  Mean ±SD or n (%) | **Pudendal block group**  **n=268**  Mean ±SD or n (%) | **Non-pudendal block group**  **n=406**  Mean ±SD or n (%) | *p*-value | **Pudendal block group**  **n=231**  Mean ±SD or n (%) | **Non-pudendal block group**  **n=102**  Mean ±SD or n (%) | *p*-value |
| **Maternal characteristics** |  |  |  |  |  |  |  |
| Age years | 32.1 ±4.1 | 31.3 ±3.9 | 31.9 ±4.0 | 0.07 | 32.3 ±4.2 | 33.6 ±4.1 | 0.21 |
| Married/cohabiting* | 951 (95.2) | 252 (95.5) | 384 (94.8) | 0.71 | 223 (97.4) | 92 (91.1) | 0.01 |
| Higher education  *Missing* | 832 (92.9)  *113* | 224 (93.7)  *29* | 327 (92.1)  *51* | 0.46 | 196 (93.3)  *21* | 85 (94.4)  *12* | 0.71 |
| Body mass index (km/m2)  *Missing* | 23.1 ±4.1  *192* | 23.3 ±4.3  *48* | 23.0 ±4.0  *87* | 0.39 | 23.2 ±4.2  *44* | 22.7 ±3.8  *13* | 0.34 |
| **Fetal characteristics** |  |  |  |  |  |  |  |
| Gestational age days (weeks) | 281 ±8.3 | 281 ±7.8 | 279 ±8.5 | <0.01 | 283 ±7.8 | 282 ±8.6 | 0.29 |
| Birth weight | 3477 ±451 | 3498 ±493 | 3400 ±419 | <0.01 | 3584 ±433 | 3480 ±452 | 0.05 |
| **Birth characteristics** |  |  |  |  |  |  |  |
| Epidural analgesia | 696 (69.1) | 139 (51.9) | 270 (66.5) | <0.001 | 193 (83.5) | 94 (92.2) | 0.04 |
| Spinal analgesia | 37 (3.7) | 17 (6.3) | 10 (2.5) | 0.01 | 7 (3.0) | 3 (2.9) | 0.97 |
| Epidural and/or spinal analgesia | 724 (71.9) | 152 (56.7) | 279 (68.7) | <0.01 | 198 (85.7) | 95 (93.1) | 0.06 |
| Episiotomy* | 480 (49.1) | 77 (29.8) | 103 (26.5) | 0.36 | 208 (90.8) | 92 (90.2) | 0.86 |
| Prolonged second stage of labor (>3 hours)* | 180 (17.9) | 33 (12.3) | 39 (9.6) | 0.27 | 84 (36.5) | 24 (23.5) | 0.02 |
| Long duration of birth (>12 hours)^1^* | 170 (17.0) | 26 (9.7) | 38 (9.4) | 0.88 | 79 (34.5) | 27 (26.7) | 0.16 |
| Oxytocin augmentation** | 612 (60.8) | 124 (46.3) | 203 (50.0) | 0.34 | 198 (86.1) | 87 (85.3) | 0.85 |
| **Pudendal block anesthetic** |  |  |  |  |  |  |  |
| Bupivacaine | 357 | 205 (82.7) | - | - | 152 (89.4) | - | - |
| Lidocaine | 50 | 35 (14.1) | - | - | 15 (8.8) | - | - |
| Bupivacaine with epinephrine | 11 | 8 (3.2) | - | - | 3 (1.8) | - | - |
| *Missing* | *81* | *20* | - | - | *61* | - | - |
| Pudendal block duration (minutes) | 67 ±61 | 71 ±58 | - | -- | 61 ±64 | - | - |
| **Birth unit**** |  |  |  |  |  |  |  |
| Unit 1 (Rikshospitalet) | 666 (66.1) | 163 (60.8) | 271 (66.7) | 0.12 | 165 (71.4) | 67 (65.7) | 0.29 |
| Unit 2 (Ullevål) | 341 (33.9) | 105 (39.2) | 135 (33.3) |  | 66 (28.6) | 35 (34.3) |  |
| **Primary outcome** | | | | | | | |
| Overt PUR*² | 327 (33.2) | 57 (21.9) | 102 (25.7) | 0.27 | 123 (53.7) | 45 (45.5) | 0.17 |
| **Secondary outcomes** | | | | | | | |
| **Residual urine volume when overt PUR was diagnosed** |  |  |  |  |  |  |  |
| ≥1000 ml^3^ | 35 (13.2) | 3 (6.0) | 14 (15.9) | 0.09 | 10 (11.1) | 8 (21.1) | 0.14 |
| ≥750^3^ | 82 (30.8) | 15 (30.0) | 28 (31.8) | 0.83 | 27 (30.0) | 12 (31.6) | 0.86 |
| Volume, ml^3^  (range) | 585 ±344  (30-1700) | 571 ±307 | 598 ±346 | 0.65 | 576 ±344 | 594 ±394 | 0.81 |
| *Missing* | *61* | *7* | *14* |  | *33* | *7* |  |
| Catheterized after >3 hours postpartum* | 116 (11.6) | 26 (9.7) | 32 (7.9) | 0.41 | 43 (18.9) | 15 (15.0) | 0.39 |
| Apgar score <7 at 5 minutes of age* | 14 (1.4) | 1 (0.4) | 4 (1.0) | 0.37 | 6 (2.6) | 3 (2.9) | 0.86 |
| Anal sphincter injury  *Missing* | 14 (1.5)  *51* | 2 (0.8)  *10* | 5 (1.3)  *20* | 0.53 | 6 (2.8)  *15* | 1 (1.0)  *6* | 0.34 |

Missing data is presented as a separate category if ≥ 5%, marked (*) if <5%, otherwise variable information was complete. ¹Vacuum and/or forceps extraction. ²Overt postpartum urinary retention (PUR): Catheterization within 3 hours postpartum. ^3^Among women diagnosed with overt PUR. ^4^T-test for continuous variables and Pearson Chi-Square for categorical variables.

**Supplementary Table 2.** Obstetric characteristic by delivery unit (n=1007)

|  | **Unit 1 (Rikshospitalet)**  **(n=666)**  Mean ±SD or n (%) | **Unit 2 (Ullevål)**  **(n=341)**  Mean ±SD or n (%) | *p*-value^3^ |
| --- | --- | --- | --- |
| Pudendal block duration (minutes) | 57 ±53 | 86 ±70 | <0.001 |
| Overt PUR¹ | 263 (40.0) | 64 (19.6) | <0.001 |
| Epidural analgesia | 485 (72.8) | 211 (61.9) | <0.001 |
| Spinal analgesia | 30 (4.5) | 7 (2.1) | 0.05 |
| Epidural and/or spinal analgesia | 508 (76.3) | 216 (63.6) | <0.001 |
| Episiotomy | 314 (48.8) | 166 (49.8) | 0.75 |
| Instrumental birth² | 232 (34.8) | 101 (29.6) | 0.10 |
| Oxytocin augmentation | 436 (65.6) | 176 (51.6) | <0.001 |

¹ Catheterization within 3 hours postpartum. ²Vacuum and/or forceps. ^3^T-test for continuous variables and Pearson Chi-Square for categorical variables. PUR=postpartum urinary retention.

**Supplementary Table 3.** Odds ratios of factors associated with overt postpartum urinary retention in spontaneous birth, stratified Unit 1

| **Unit 1 (Rikshospitalet)**  **n=410²** | | | | | | | | |
| --- | --- | --- | --- | --- | --- | --- | --- | --- |
|  | Unadjusted OR | 95% CI | | *p*-value | Adjusted OR^3^ | 95% CI | | *p*-value |
| **Pudendal nerve block** | **0.72** | **0.47** | **1.12** | **0.14** | **0.65** | **0.41** | **1.03** | **0.07** |
| Epidural/ spinal analgesia | 2.26 | 1.39 | 3.69 | <0.01 | 2.25 | 1.35 | 3.75 | <0.01 |
| Episiotomy | 1.47 | 0.92 | 2.35 | 0.11 | 1.36 | 0.84 | 2.19 | 0.21 |
| **Unit 2 (Ullevål)**  **n=220²** | | | | | | | | |
|  | Unadjusted OR | 95% CI | | *p*-value | Adjusted OR^3^ | 95% CI | | *p*-value |
| **Pudendal nerve block** | **1.47** | **0.67** | **3.20** | **0.33** | **1.67** | **0.75** | **3.72** | **0.21** |
| Epidural/ spinal analgesia | 1.57 | 0.69 | 3.54 | 0.28 | 1.81 | 0.78 | 4.19 | 0.17 |
| Episiotomy | 1.18 | 0.52 | 2.69 | 0.70 | 1.23 | 0.53 | 2.84 | 0.62 |
|  | | | | | | | | |
|  |  |  | |  |  |  | |  |
|  |  |  |  |  |  |  |  |  |
|  |  |  |  |  |  |  |  |  |
|  |  |  |  |  |  |  |  |  |

¹Vacuum and/or forceps extraction. **²**Number of women included in the analysis. ^3^Multivariable model adjusted for epidural/spinal analgesia and episiotomy. OR: Odds ratio; CI: Confidence interval.
